# Indications for Lancing the Gums

**Published:** 1871-04

**Authors:** 


### ARTICLE VII.

### *Indications for Lancing the Gums.*

It is but a short time since I sat at the feet of learned professors in a medical college. It seems, therefore, a little presumptuous for one who is but just getting his own wisdom teeth, in the things of our art, to write an article for a medical journal. Yet perhaps its readers, in their clemency, will bear with me a little.

One of our professors, I well remember (and he was my ideal of a lecturer), in expatiating on the effectiveness of the homœopathic remedy in almost every condition of human suffering, took occasion to denounce the practice of lancing gums in difficult dentition. He said considerable on the subject, the sum of which was, that in true homœopathic practice, lancing was *never* called for.

Now it so happened that I grew up on the farm, and, as the lecture went on, I bethought myself that I knew something about the young of animals, if I did not of babies.

So, at the risk of a little chuckle at my own expense, I made bold to remark, that, in tending my father's flocks, I had, I thought, saved many a lamb by timely cutting its gums. I had often found them prostrate on the ground, frothing at the mouth, moaning and refusing all nourishment. A little examination would show a thin, tough glistening membrane, drawn tightly across the incisor teeth; cutting this seemed to give almost instantaneous relief. The lamb would at once take to its mother, and soon be as lively as its fellows.

I remarked further, that it was well known among farmers that young colts often died for want of a little assistance in hastening the eruption of backward teeth.

Well, I drew upon myself what I expected—a little laugh, and many a look which said, you had better have kept still.

But, to continue: Now that I am having a little practice in caring for babies, I must give you a bit of experience in that line.

A few nights since I was called, in great haste, to see a child that just had a fit. I found the child was having spasms. I administered the homœopathic dose according to the symptoms as they appeared to me, and I thought with some success. The patient had no more spasms for a number of hours. But the paroxysms returned. I applied myself diligently, seeking counsel from Guernsey, Raue, Lippe and others, that, if *possible*, I might get the remedy that was *exactly* homœpathic to the condition. But the spasms kept coming more and more frequent, and harder, and it did seem as if the child must die. The parents gave it up. But finally, as it came out of a terrible spasm, and just as it was going into another, I went for the little tooth, which was pressing hard against a glistening tough membrane. The operation was soon done, *and the little sufferer was just as soon relieved.* It went right to sleep, and slept sweetly for half an hour, and it has not had another spasm yet.

Now, of course, I can but blame myself for not using the lancet sooner. But how much of blame rightly belongs to that professor for the influence his lecture had on me, I

leave for others so judge. Hereafter I shall profit from my early surgical experience with the lambs, and *especially* from this recent case in the baby. I shall, however, make a distinction between retarded dentition and difficult dentition; that is, if I find the teeth of sufficient length to protrude above the gums, and yet pressing hard against an unruptured covering, I shall do a little cutting as well as a little dosing. All honor to the single homœopathic remedy, and to the high potency, too, if you please. I believe in both; but hereafter shall endeavor not to be *too* conservative in the use of the knife or in the use of any mechanical interference that may best meet the wants of the given case.—*Med. Investigator*.

---

